# Comparison of cognitive ability and its distribution between men with autism spectrum disorder and attention-deficit/hyperactivity disorder

**DOI:** 10.1371/journal.pone.0345522

**Published:** 2026-03-27

**Authors:** Hirokazu Doi, Yoshifumi Nakamura, Ayaka Nakai, Chieko Kanai, Haruhisa Ohta

**Affiliations:** 1 Department of Information and Management Systems Engineering, Nagaoka University of Technology, Kamitomioka Nagaoka, Niigata, Japan,; 2 Department of Psychiatry, Showa University, Japan,; 3 Faculty of Humanities, Wayo Women’s University, Japan,; 4 Medical Institute of Developmental Disabilities Research, Showa University, Japan; Liverpool John Moores University, UNITED KINGDOM OF GREAT BRITAIN AND NORTHERN IRELAND

## Abstract

**Objectives:**

Clarification of the strengths and weaknesses of cognitive ability is essential to our understanding of the characteristics of autism spectrum disorder and attention deficit/hyperactivity disorder. However, whether individuals with these conditions exhibit distinct patterns of cognitive ability remains unclear. To address this point, we aimed to compare the cognitive profiles of patients with autism spectrum disorder with those of patients with attention deficit/hyperactivity disorder by placing special emphasis on the distribution of cognitive function within each group.

**Methods:**

This study compared the Wechsler Adult Intelligence Scale index scores of men with autism spectrum disorder and attention deficit/hyperactivity disorder. A machine learning model was trained to classify autism spectrum disorder and attention deficit/hyperactivity disorder based on the subtest scores. The conformity of the within-group distribution of each index score to a normal distribution was also tested.

**Results:**

Individuals with autism spectrum disorder scored higher than those with attention deficit/hyperactivity disorder in the Verbal Comprehension Index and Working Memory Index, while the opposite pattern was observed for the Perceptual Organization Index. The classification performance of the machine learning model was above chance level. The distributions of the Verbal Comprehension Index and Perceptual Organization Index deviated significantly from a normal distribution only in the autism spectrum disorder group. The results of Gaussian mixture clustering indicated that men with autism spectrum disorder could be divided into two distinct clusters based on their Verbal Comprehension Index scores.

**Conclusions:**

Our findings indicate that men with autism spectrum disorder and attention deficit/hyperactivity disorder show distinct cognitive profile patterns from each other. The distribution of some of the index scores deviated from the normal distribution only in autism spectrum disorder, which supports the view that autism spectrum disorder comprises heterogeneous subgroups with different cognitive profiles.

## 1. Introduction

Autism spectrum disorder (ASD) and attention-deficit/hyperactivity disorder (ADHD) are among the most prevalent neurodevelopmental conditions, with prevalence rates of approximately 8% for ADHD [1] and 3% for ASD [2]. People with ASD generally exhibit persistent deficits in social communication and interaction along with restricted interests and sensory issues [3], whereas individuals with ADHD commonly present with persistent inattention and hyperactivity [3]. It is generally accepted that ASD and ADHD are hereditary conditions. However, several studies have also suggested the contribution of environmental factors [4–6]. Thus, the etiologies of ASD and ADHD remain unclear.

Understanding the strengths and weaknesses of cognitive function in individuals with neurodevelopmental disorders is essential for developing effective intervention strategies [7]. People with ASD and ADHD often demonstrate atypical functioning patterns across various cognitive domains beyond social and communicative abilities. Numerous studies have reported poorer performance in behavioral tasks assessing executive function [8] and working memory [9,10] in individuals with ASD and ADHD than in their neurotypical counterparts. However, individuals with ASD have also demonstrated superior performance in specific tasks such as detecting local patterns embedded within Figs [11,12].

People with ASD and ADHD share many commonalities in their symptoms and behavioral phenotypes [13]. However, whether ASD and ADHD show distinct cognitive profile patterns from each other remains uncertain [7]. Clarification of this point is important from the standpoint of clinical practice because it is relevant to the usefulness of cognitive assessment in the diagnosis of these conditions [7]. However, much of the previous research has compared the cognitive functions of these clinical populations with those of their typically developing counterparts, making it difficult to explore group differences between conditions.

Several previous studies [14–17] have directly compared the cognitive profiles of patients with ASD and ADHD across various age ranges. A recent meta-analytical review [7] identified several potential and notable differences in the cognitive profiles of patients with ASD and ADHD. However, the number of relevant studies was not large, especially for adults, and the sample size of each study was relatively small [7]. Moreover, the detailed patterns of differences in the cognitive profiles of patients with ASD and ADHD were inconsistent among the studies.

The behavioral phenotypes of individuals with ASD are notably diverse or heterogeneous [18,19], and it is widely accepted that autism exists on a spectrum that encompasses individuals with varying symptoms. Furthermore, ASD includes subgroups such as autism and Asperger’s syndrome, which have been removed from the DSM-5. Similarly, ADHD can be classified into predominantly inattentive, predominantly hyperactive-impulsive, and combined types [3].

Gaining comprehensive insight into the nature of such heterogeneity in cognitive function among individuals with ASD and ADHD is crucial to improving our understanding of these conditions. Given the reported heterogeneity in these groups, especially in ASD [18,19], there is a high chance that the distribution of cognitive abilities deviates from the normal distribution within each condition, in contrast to the general assumption of normality. This can occur under several scenarios. First, categorically distinct subgroups may be identified in the distribution of cognitive function. In this case, the overall distribution could be approximated by summing multiple normal distributions. Second, if the proportion of participants with extremely low or high index scores is relatively large, the tails of the distribution become thicker than expected under a normal distribution, and vice versa, as captured by the kurtosis of the distribution.

Analysis of the distribution provides valuable insights into the nature of the heterogeneity in cognitive abilities within each group. However, many existing studies on cognitive function in ASD and ADHD have focused only on group means, resulting in relatively little attention being paid to the distribution of cognitive abilities within each condition.

To address this knowledge gap, we compared the cognitive functions of these groups, with special emphasis on the within-diagnosis distribution of cognitive abilities. The novelty of our approach lies in the analysis of the distribution of cognitive abilities, which provides information on the heterogeneity in each aspect of cognitive ability within each condition. In addition to comparing group differences in cognitive ability, the usefulness of cognitive function in identifying ASD and ADHD was explored using multivariate analysis [17]. A machine learning model was trained to classify ASD and ADHD based on indices of multiple aspects of cognitive function, and its classification performance was evaluated.

The cognitive abilities of men with ASD or ADHD were evaluated using the Wechsler Adult Intelligence Scale (WAIS) [20], a standardized test battery. The WAIS offers several advantages in cognitive evaluation. First, it consists of multiple tests designed to assess abilities across various cognitive domains, providing a comprehensive cognitive profile for each individual [20]. Second, it is designed for use across a wide range of populations with diverse backgrounds, including individuals with ASD and ADHD [15,21,22], enabling valid comparisons of cognitive profiles across diagnostic groups.

In this study, we used the Wechsler Adult Intelligence Scale version III (WAIS-III) dataset [20] because it has been in clinical use for approximately 15 years in Japan, and we had established a large dataset of index scores. Numerous studies have used the WAIS-III, and the characteristics of each index score within the WAIS-III are well-documented in various populations. Thus, although it has now been replaced by the WAIS-IV and WAIS-V, the data obtained using the WAIS-III dataset provided adequate information on the cognitive abilities of people with ASD and ADHD.

ASD and ADHD are overrepresented in men [23,24]. The amount of data for women with ASD and ADHD in our dataset was relatively limited, which made the estimated distribution of the index scores unreliable. Thus, we analyzed the cognitive profiles of men in this study.

## 2. Method

### 2.1. Participants

This retrospective study using an opt-out method was conducted in accordance with the Declaration of Helsinki, and approved by the ethical committee of the faculty of Medicine, Showa University (Approval No. B-2016–029). The participants were outpatients of Karasuyama Hospital, Showa University, Tokyo, Japan. Patients were diagnosed with ASD, including Asperger’s syndrome, autism, and pervasive developmental disorder not otherwise specified (PDD-NOS), or ADHD, by certified clinicians based on the diagnostic criteria of the DSM-IV-TR or DSM-5, following a consensus by a team of experienced psychiatrists. The diagnostic criteria changed from DSM-IV-TR to DSM-5 while the WAIS-III was still in use in Japan. Therefore, to retain as many samples as possible, we aggregated the data from participants diagnosed under different versions of the DSM into the final dataset. Patients diagnosed with comorbid ASD and ADHD based on the DSM-5 were discarded from the final sample. The WAIS-III [20] was administered by licensed psychologists, at the time of the participants’ initial examinations, to obtain their cognitive profiles. Data from participants aged 20–50 years at the time were included in the analysis. The final sample consisted of 516 and 206 participants with ASD and ADHD, respectively. Comorbidities included intellectual disabilities (8 ASD, 4 ADHD), mood disorders (12 ASD, 11 ADHD), anxiety disorders (2 ASD, 0 ADHD), obsessive-compulsive disorders (8 ASD, 0 ADHD), schizophrenia (2 ASD, 1 ADHD), Tourette’s disorder (1 ASD, 1 ADHD), and epilepsy (2 ASD, 1 ADHD). A total of 293 men with ASD and 82 men with ADHD already had a history of medication use at the time of the initial examination. A Brunner–Munzel test revealed no significant inter-group difference in age (*statistic* = 1.12, *p* = 0.26; M = 28.8 years, SD = 7.4 for ASD; M = 29.8, SD = 8.1 for ADHD). The Brunner–Munzel test also revealed no significant inter-group differences in full-scale IQ (FSIQ) (*statistic* = 0.236, *p* = 0.813).

### 2.2. Measurement of cognitive abilities

Participants’ cognitive abilities were assessed using the WAIS-III test battery [20]. The WAIS-III quantifies four index scores: the Verbal Comprehension Index (VCI), Working Memory Index (WMI), Perceptual Organization Index (POI), and Processing Speed Index (PSI). These index scores were calculated based on the performance across multiple subtests: vocabulary, information, and similarities for the VCI; arithmetic, letter-number sequencing, and digit span for the WMI; block design, matrix reasoning, and picture completion for the POI; and digit symbol coding and symbol search for the PSI. Comprehension, picture arrangement, and object assembly scores were also collected.

### 2.3. Analysis

#### 2.3.1. Group comparison of index scores.

We focused on the analysis of the index scores, specifically VCI, WMI, POI, and PSI. In the first set of analyses, we analyzed group differences in each index score between patients with ASD and ADHD using the Brunner–Munzel test. Four comparisons were made. Thus, the significance threshold was adjusted to 0.05/4 = 0.0125 using Bonferroni correction to guard against the inflation of the false-positive rate.

The effect of diagnosis was further explored using quantile regression [25] for the 50% quantile, which does not assume normality in the response variable, with each index score as the response variable and diagnosis, age, and FSIQ as predictors to control for the effects of age and FSIQ.

#### 2.3.2. Analysis of subtest scores by group comparison and machine learning approach.

The scores of the subtests that constituted the index scores for the ASD and ADHD groups were compared using the Brunner–Munzel test. This was an explorative analysis because we did not have any specific hypotheses regarding group differences in the subtests. Therefore, we did not apply the significance threshold adjustment.

The informativeness of subtest scores was further explored using a machine learning approach [17,26]. In this analysis, a random forest classifier was trained to classify ASD and ADHD based on the subtest scores. The random forest classifier is an ensemble learning algorithm in which the final classification is determined by the majority voting of multiple weak tree classifiers [27]. The number of estimators and the maximum depth of the weak classifier were set to 100 and 3, respectively. The classification performance was evaluated using 5-fold stratified cross-validation. The main performance indicator was the area under the curve (AUC) of the receiver-operator-characteristic (ROC) curve. The 95% confidence interval (CI) of the ROC curve was estimated using non-parametric bootstrap sampling, *i.e.,* the random resampling with replacement method, with 1,000 iterations. We employed a non-parametric bootstrapping approach because no assumptions could be made regarding the form of the underlying distribution. The optimal classification threshold was determined using the Youden Index. The performance metrics, *i.e.,* accuracy, precision, recall, F1 and specificity, were computed at this threshold.

#### 2.3.3. Analysis of within-diagnosis distribution of index scores.

We analyzed the shape of the within-diagnosis distribution for each index score. The normality of the distribution of the index scores within each group was then tested. Normality tests were conducted for each of the four indices. Therefore, the significance threshold was adjusted to 0.05/4 = 0.0125 using Bonferroni correction.

If a distribution significantly deviated from normality, its characteristics were further explored in two ways: Deviations in kurtosis and skewness from the normal distribution (excess kurtosis and skewness = 0) were examined using kurtosis [28] and skewness tests [29]. A positive excess kurtosis indicates a leptokurtic distribution with a sharper peak than a normal distribution, whereas a negative kurtosis indicates a platykurtic distribution with a flattened, less-peaked shape. Positive skewness indicates a shift in the mass of the distribution towards the lower range, whereas negative skewness indicates a shift towards the higher range.

To determine whether the distribution was composed of multiple Gaussian distributions, the frequency distribution was subjected to Gaussian Mixture Modelling (GMM). The optimal number of clusters was determined using the Bayesian Information Criterion (BIC), with the number of clusters corresponding to the minimal BIC value considered to be the optimal number of clusters.

## 3. Results

### 3.1. Group difference in index scores

**Fig 1:**
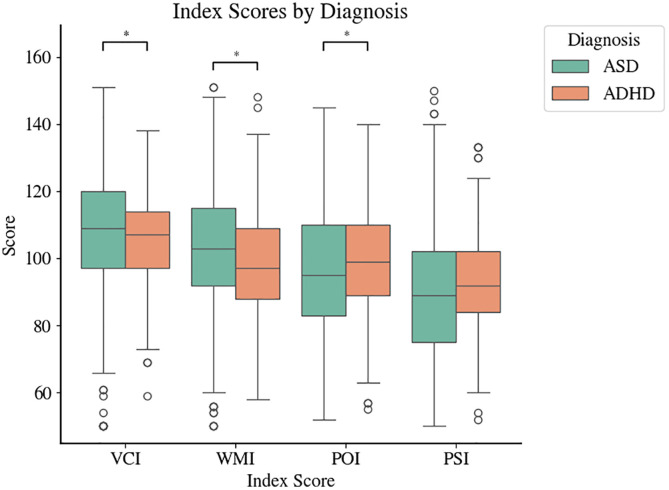
Boxplots of the index scores in ASD and ADHD. *p < 0.05.

Boxplots of the four index scores for each group are shown in Fig 1. Significant between-group differences were observed in VCI (*statistic* = −2.57, *unadjusted p* = 0.01), WMI (*statistic* = −2.97, *unadjusted p* = 0.003), and POI (*statistic* = 2.55, *unadjusted p* = 0.011) but not in PSI (*statistic* = 2.25, *unadjusted p* = 0.025). Quantile regression analysis revealed a significant effect of diagnosis on VCI (*Coefficient* = 3.752, *t* = 3.987, *unadjusted p* < 0.001), WMI (*Coefficient* = 3.452, *t* = 2.747, *unadjusted p* = 0.006), and POI (*Coefficient* = −3.42, *t* = −3.023, *unadjusted p* = 0.003). However, the effect of diagnosis for PSI was not significant (*Coefficient* = −1.605, *t* = −1.09, *unadjusted p* = 0.276).

### 3.2. Group difference in subtest scores

The subtest scores constituting the four index scores for the ASD and ADHD groups were compared. The results are summarized in Table 1.

**Table 1:** The 25%, 50%, and 75% quantiles of the scores of the subtests in ASD and ADHD.

Index Score	Subtest	ASD			ADHD			*Statistics*	*unadjustedp value*
		Q1	Q2	Q3	Q1	Q2	Q3		
**VCI**	**Information**	9	12	14	7.75	10	12	−4.477	<.001**
	**Similarities**	9	12	14	9	11	14	−1.195	0.233
	**Vocabulary**	10	12	14	10	12	14	−0.688	0.492
**WMI**	**Arithmetic**	9	11	14	8	11	12	−2.971	0.003**
	**Digit Span**	8	10	13	8	10	12	−1.831	0.068#
	**Letter-Number-Sequencing**	8	10	12	7	9	11	−2.576	0.01*
**POI**	**Block Design**	6	10	13	7	10	12	0.483	0.629
	**Matrix Reasoning**	9	11	13	10	12	13	2.316	0.021*
	**Picture Completion**	5	8	10	6	9	11	4.224	<.001**
**PSI**	**Digit Symbol Coding**	5	8	10	6	8	10	0.985	0.325
	**Symbol Search**	6	8	11	7	9	12	2.987	0.003**

Q1, Q2, and Q3 represent the 25%, 50%, and 75% quantiles, respectively. # p < 0.10, *p < 0.05, **p < 0.01. A significance threshold adjustment was not applied because of the exploratory nature of the subtest analysis.

**Fig 2:**
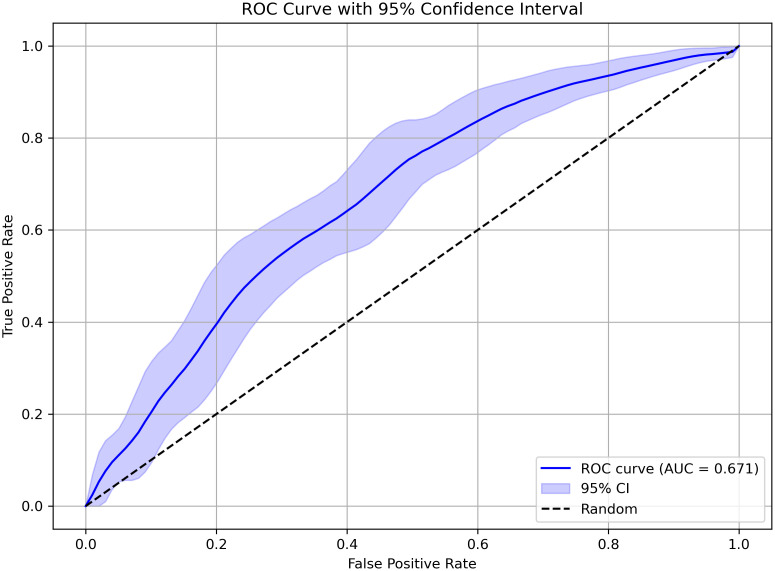
ROC curve with 95% confidence interval. The blue line and the band represent the averaged ROC curve and the 95% CI obtained using bootstrap sampling.

The scores on all subtests were available for some participants (*n* = 406 for ASD, *n* = 170 for ADHD). The subtest scores of these participants were used to train a random forest model to classify ASD and ADHD. The ROC is shown in Fig 2. The AUC was 0.671. Although the classification performance was modest, the lower limit of the 95% CI was above the diagonal line, indicating that the classification performance level was above chance. The metrics at the optimal threshold, as determined using the Youden Index, were accuracy = 68.1%, precision = 0.79, recall = 0.74, specificity = 0.54, and F1 score = 0.765.

### 3.3. Analysis of distribution of index scores

**Fig 3:**
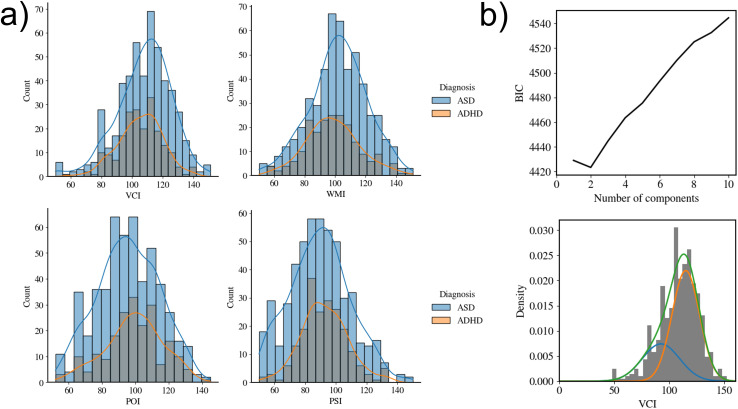
a) The frequency distribution of each index score for ASD and ADHD. The curve is the density distribution estimated using kernel density estimation. b) **Upper Panel:** BIC at each number of clusters, **Lower Panel:** Underlying clusters revealed using GMM. Blue and orange curves represent the density distribution of the underlying clusters. The green curve is the summation of the blue and orange curves. The gray histogram represents the frequency distribution for the VCI. The difference in the overall shape of the histogram from Fig 3-a) is due to differences in the bin settings.

The frequency distribution of each index score for each group is shown in Fig 3a. In the ASD group, a normality test revealed that the distributions of the VCI (*statistic* = 29.89, *unadjusted p* < 0.001) and POI (*statistic* = 10.09, *unadjusted p* = 0.006) deviated significantly from normality. No index score distribution deviated significantly from normality in the ADHD group.

The results of the kurtosis and skewness tests for the VCI and POI are summarized in Table 2. The kurtosis and skewness tests were each conducted four times. Therefore, the significance threshold was adjusted to 0.05/4 = 0.0125 using the Bonferroni method. The VCI distribution exhibited significantly negative skewness, whereas the POI distribution was platykurtic, with significantly negative kurtosis in the ASD group. Using GMM, the optimal number of clusters was determined to be two for the VCI and one for the POI. The underlying VCI distributions are shown in Fig 3b.

**Table 2:** Kurtosis and Skewness in VCI and POI in each group.

Index Score	Diagnosis	Kurtosis			Skewness		
		*Value*	*Statistic*	*unadjusted* *p-value*	*Value*	*Statistic*	*unadjusted* *p-value*
**VCI**	**ASD**	0.596	2.366	0.018	−0.559	−4.929	<.001**
	**ADHD**	0.133	0.621	0.5345	−0.285	−1.699	0.0894
**POI**	**ASD**	−0.52	−3.17	0.0015**	−0.021	−0.201	0.8404
	**ADHD**	−0.227	−0.539	0.5901	−0.247	−1.478	0.1395

**p < .01 after significance threshold adjustment.

The characteristics of the two clusters identified in the VCI distribution were explored further. The mean and standard deviation of the VCI in the two identified clusters are summarized in Table 3, with the mean VCI being higher in Cluster 1 than in Cluster 2. Information on years of schooling was available for some participants, but not all. The Brunner–Munzel test revealed that the number of years of schooling was significantly longer (*statistics* = 5.26, *p* < 0.0001) in Cluster 1 (M = 15.6, SD = 1.85 years, *n* = 278) than in Cluster 2 (M = 14.3, SD = 1.87 years, *n* = 73).

**Table 3:** Mean and standard deviation (SD) of VCI and the number of participants in each cluster.

	Cluster 1	Cluster 2
**Mean**	115.2	83.4
**SD**	11.2	11.3
**N**	397	119

N represents the number of participants in each cluster.

A significant proportion of participants with ASD were diagnosed with Asperger’s syndrome, autism, and PDD-NOS based on the diagnostic criteria of the DSM-IV-TR. The number of participants with these three diagnoses is summarized in Table 4. In both clusters, nearly half of the participants were classified as having PDD-NOS. Most of the remaining participants in Cluster 1 had Asperger’s syndrome whereas those in Cluster 2 had autism. The chi-squared test revealed a significant difference in the distribution of the diagnoses between the clusters (χ^2^ = 15.5, *p* < 0.001).

**Table 4:** Distribution of Asperger’s syndrome, autism, and PDD-NOS in each cluster.

	Asperger’s	Autism	PDD-NOS	Total
**Cluster 1**	67	33	85	185
	(36.2)	(17.8)	(45.9)	
**Cluster 2**	5	18	21	44
	(11.4)	(40.9)	(47.7)	

In parenthesis are the within-cluster proportions (%) of each diagnostic group.

## 4. Discussion

### 4.1. Distinctive cognitive profile between ASD and ADHD

We investigated differences in the WAIS index and subtest scores of patients with ASD and ADHD, with a particular focus on differences in the distribution of index scores based on a large dataset. Overall, men with ASD and ADHD showed distinct patterns of cognitive function. Specifically, significant group differences were observed in the VCI, WMI, and POI but not the PSI. The distributions of the VCI and POI deviated significantly from normality in the ASD group, but not in the ADHD group. The random forest classifier succeeded in classifying ASD and ADHD based on the subtest scores with above chance level performance, which supports the view that cognitive profiles could help classify these conditions.

### 4.2. Group difference in WMI

Group comparisons revealed a higher WMI in the ASD group than in the ADHD group. Group comparisons of the subtests revealed statistically significant or marginally significant group differences in the three subtests constituting the WMI, indicating that people with ASD perform better than those with ADHD in multiple aspects of working memory.

Working memory is considered a core component of executive function [30,31], enabling the execution of planned behavior through the maintenance, updating, and manipulation of relevant information [32,33]. Numerous studies have reported poorer performance in working memory and related executive control tasks in patients with ASD and ADHD than in their typically developing counterparts [9,10]. However, studies on pediatric samples have reported relatively preserved working memory abilities in patients with ASD [34,35]. A later study [35], which reanalyzed published data on executive function in children with ASD reported that only a small proportion of children with ASD show deficits in behavioral executive control. Additionally, a direct comparison between ASD and ADHD indicated that individuals with ASD showed greater improvements in executive function task performance over time than those with ADHD [36]. Thus, the relatively preserved working memory ability and its developmental improvement likely explain our finding that men with ASD outperform those with ADHD in working memory tasks. However, the WMI of the WAIS is not a pure measure of working memory because its results can be influenced by factors beyond working memory capacity [37,38]. Therefore, our findings should be validated using cognitive tasks specifically designed to assess working memory under more stringent experimental conditions.

### 4.3. Group difference in PSI

In the current dataset, no significant difference in the PSI was observed between the ASD and ADHD groups. Two previous large-scale studies have also investigated the clinical significance of PSI in patients with ASD and ADHD. Mayes and Calhoun (2007) [39] reported that children with ASD and ADHD performed worse on processing speed tasks than typically developing children (see also [40]); however, no significant differences were observed between ASD and ADHD. A later study [41] identified a specific association between inattentiveness and PSI scores. Our recent findings are in line with those of Mayes and Calhoun (2007) [39], and are consistent with the view that a slow processing speed is not specifically associated with either ASD or ADHD.

### 4.4. Group difference in VCI

Group comparisons revealed a higher VCI in the ASD group than in the ADHD group. Analysis of the subtests indicated that this observation was driven mainly by group differences in the information subtest. The information subtest in the WAIS-III measures general knowledge about people, places, and objects [42]. Ghaziuddin and Gerstein (1996) [43] reported that people with ASD often have an overly “pedantic” style of speech, which includes the conveyance of excessive information. Such attentiveness towards detailed information or knowledge in people with ASD might be reflected in their superior performance in the information subtest.

The distribution of the VCI deviated significantly from normality. Further analyses suggested that the VCI distribution might be caused by the summation of two overlapping normal distributions. This finding indicates that men with ASD can be categorized into two distinct groups based on their linguistic abilities. The ASD group outperformed the ADHD group in the VCI, likely due to the presence of a high VCI subgroup within the ASD sample. Further characterization of each cluster revealed significantly longer years of schooling in clusters with higher VCI. This finding is consistent with that of a recent study using the WAIS-IV, which revealed an association between educational background and the VCI in individuals with ASD [44]. The proportion of patients with Asperger’s syndrome was larger than that of patients with autism in Cluster 1, but the opposite was true in Cluster 2. This observation is not surprising because the diagnostic criteria for Asperger’s syndrome include a lack of clinically significant language delay [45]. The boundaries of the clusters also did not align with the distinction between Asperger’s syndrome and the other subcategories of ASD. Nearly half of the members in both clusters had PDD-NOS. Therefore, further research is required to elucidate the predispositions that engender categorical differences in VCI in people with ASD.

Two studies recently identified subgroups or clusters of linguistic ability in individuals with ASD. Schaeffer et al. (2023) [46]. highlighted the existence of at least three subgroups of linguistic ability, characterized by structural language ability and verbal expressiveness. Similarly, Song et al. (2022) [47] identified four subgroups of children with ASD based on their linguistic ability using an unsupervised learning approach. In their classification, linguistic ability covaried with intelligence and the severity of autism symptoms. How the subgroups identified in the present study correspond with those reported in previous studies [46,47] remains unclear.

Recent studies [48,49] have raised concerns that GMM may falsely detect spurious clusters in multidimensional cognitive data, particularly when the assumption of orthogonality across dimensions is violated. In the current study, clusters were derived from unidimensional VCI scores to avoid the problem of non-orthogonal dimensions. Nevertheless, given the relatively small sample size and exploratory nature of the analysis, the robustness of the clustering results should be interpreted with caution.

### 4.5. Group difference in POI

The ADHD group outperformed the ASD group in terms of POI, which reflects visuospatial organization and visuomotor skills [20]. An atypical pattern of perceptual organization has often been reported in patients with ASD. This atypicality is commonly described as “weak central coherence” [50,51], referring to a reduced ability to integrate elements into a coherent whole, and an increased focus on and enhanced processing of details [11]. Gambra et al. (2024) [51] reported that children with ASD, but not those with ADHD, exhibited poorer performance in central coherence tasks than typically developing controls. Our current findings extend these results [51] by demonstrating that the weaker central coherence observed in patients with ASD persists into adulthood.

Among the subtests constituting the POI, children with ASD exhibited poorer performance than those with ADHD in matrix reasoning and picture completion. However, the performance of the block design subtest was comparable between the two groups. Interestingly, the interquartile range for block design in ASD was the largest among all subtests explored in the present study. These findings indicate relatively spared performance at the group level and large individual differences in the block design task in children with ASD. An influential study [52] reported better performance in people with ASD in completing the block design task than typically developed controls. However, the results of later studies were inconsistent (for a brief review, see [12]), which may be explained by the relatively large individual differences in this task, as observed in the current study.

The distribution of POI scores in the ASD group deviated significantly from the normal distribution with pronounced negative kurtosis, indicating that the distribution was flatter and less peaked than expected for a normal distribution. To our knowledge, this is the first study to report a deviation from the normal distribution of POI scores in patients with ASD. One potential explanation for this is that the large individual differences in the block design task contributed to the flattened peak of the distribution. However, this is merely speculation, and future studies should aim to replicate and elucidate the cause of these findings using intelligence test batteries and cognitive tasks conceptually related to POI.

### 4.6. Limitations

This study had several limitations. First, the effects of comorbidities were not actively controlled in this set of analyses. Participants diagnosed using the DSM-IV diagnostic criteria were included to increase the sample size. Consequently, we cannot exclude the possibility that the ASD group in the current sample included individuals with comorbid ADHD, which may have obscured group differences in the cognitive abilities of ASD and ADHD, and in turn, reduced the performance of the random forest model in classifying the two groups. In addition to the elimination of ASD subcategories and the recognition of comorbidities in ASD and ADHD in DSM-5, it cannot be ruled out that participants diagnosed under DSM-IV and DSM-5 differ systematically in their characteristics and attributes due to changes in other aspects of the diagnostic criteria, such as the relaxation of the age-of-onset criteria [3]. This issue should be addressed by recruiting an adequate number of participants diagnosed exclusively based on DSM-5 criteria.

In addition, a substantial proportion of the participants in this study already had a history of medication use at the time of WAIS administration and had comorbid psychiatric conditions. Symptoms of mood disorders have been shown to affect WAIS index scores [53,54], especially PSI [41,53]. A closely related study [22] reported that the cognitive profile measured using the WAIS-III in adults with ADHD was influenced by comorbidities of ASD and depression. Speculation of how these confounders may have influenced the present findings is difficult at this point because of the scarcity of relevant knowledge reported in previous studies. The exclusion of confounders such as comorbidities and medication is an important topic for future studies.

Second, the sample size was relatively small. Therefore, although we did not find any signs of deviation from the normal distribution in the ADHD group, we cannot draw firm conclusions regarding the distribution of the index scores in this group. Similarly, no significance threshold adjustment was applied to the group comparison of the subtests owing to the explanatory nature of this analysis. In conjunction with the relatively small sample size, the significant group differences detected in the subtests should be interpreted with caution because they could easily be a result of a Type I error.

Third, the age at which participants underwent the WAIS-III varied widely, ranging from young adulthood to early senescence. The number of longitudinal studies on the stability of index scores remains limited, and their findings are sometimes contradictory [55,56]. For example, Murray et al. (2017) [56] reported stability in the WISC scores from childhood to adolescence in individuals with ADHD. Conversely, another study [55] detected a slight decrease in verbal comprehension and processing speed scores in children with ADHD at roughly the same developmental stages. A recent ASD study [57] detected a significant increase in PSI and POI scores at the individual level after 18 years of age, whereas VCI and WMI remained stable. Therefore, whether the present findings hold when participants are stratified by age at the time of test administration remains to be determined.

Fourth, the present study included only men because of the relatively small number of women in the current dataset, which limits the generalizability of the findings. Women with ASD often “camouflage” their symptoms using adaptive behaviors [58]. Consequently, women with ASD and higher intellectual abilities are more likely to be overlooked or diagnosed later in life [59].

Given the underrepresentation of women with ASD, particularly within the high-IQ range [59], it is conceivable that the distribution of index scores may differ between men and women with ASD. This may be especially relevant for the VCI and WMI included in verbal IQ. Using a sample similar to that of the present study, Doi et al. (2022) [17] reported significantly higher verbal IQ, but not performance IQ, in men with ASD than in women with ASD. Furthermore, a related study [59] demonstrated that a larger proportion of men with ASD exhibited an IQ discrepancy in favor of verbal IQ over performance IQ compared with women with ASD. The same study [59] found generally higher scores on subtests contributing to verbal IQ, particularly the information subtest, in men with ASD than in women with ASD.

As is the case with ASD, women with high cognitive abilities are less likely to be diagnosed with ADHD during childhood [60] as they often compensate for symptoms through coping strategies [61]. Research on sex differences in cognitive profiles among individuals with ADHD remains limited; however, existing studies suggest nuanced patterns of sex differences in executive function and working memory [62]. Therefore, whether the present observations can be generalized to women with ADHD remains uncertain.

Finally, the present findings are based on an analysis of large-scale data collected using the WAIS-III. Whether the same results can be replicated using a newer version of the WAIS is an empirical question. Whether the present results hold after revision of the subtests included in the computation of each index score is also of interest. As stated above, concerns have been raised regarding the validity of several subtests used to measure cognitive profiles. Several studies have pointed out that the arithmetic subtest included in the WMI is influenced by basic mathematical skills rather than working memory alone [37,38]. In addition, several studies have reported mathematical difficulties in individuals with ADHD, even without the comorbidity of dyscalculia [63], indicating the differential sensitivity of this subtest across neurodevelopmental conditions. Likewise, it is highly likely that performance on the information subtest in the VCI reflects cultural exposure and educational background rather than linguistic ability itself. Moreover, the arithmetic and information subtests, which were originally core subtests in the WISC-III, have been reclassified as supplementary subtests in the WISC-IV [64].

All things considered, the results for the VCI and WMI in the present study may have been unduly affected by the specific subtests included in the calculation of these index scores. The constructs measured by the WAIS are generally considered relatively stable across different versions of the test [65]. However, given the aforementioned empirical considerations and the possibility of future subtest revisions, ensuring that the present results can be replicated using different versions of the WAIS is essential.

## 5. Practical implications

The group comparison indicated that men with ASD and ADHD exhibit distinct patterns of cognitive profiles from each other, which is consistent with the findings of several previous studies [7,15–17]. Nevertheless, the classification performance of the random forest classifier in the present study was only modest, although statistically above chance. Overall, the results support the usefulness of cognitive assessment in characterizing differences in the cognitive abilities of men with ASD and ADHD. However, they also highlight the limitations of cognitive profiles as a diagnostic tool for distinguishing between these two conditions, in line with a recent meta-analysis that has cast doubt on the specificity of the WAIS [7]. The inclusion of other validated indices of cognitive function as features may improve the classification performance and change the current situation.

Interestingly, the present study, along with several closely related ones [46,47], raises the possibility that subgroups of individuals with ASD can be identified using unsupervised learning based on cognitive function data. However, this research is still in the early stages. Nevertheless, the detection and validation of such subgroups in a data-driven manner based on cognitive function characteristics may lead to the development of tailored intervention strategies.

## 6. Conclusions

This study examined the inter-group differences and distribution patterns of cognitive abilities in men with ASD and ADHD. Group comparisons revealed significant differences in VCI, WMI, and POI but not in PSI, supporting the distinctiveness of cognitive abilities in men with ASD versus ADHD. A machine learning model trained to distinguish ASD from ADHD based on cognitive profiles demonstrated only modest performance, indicating that the diagnostic usefulness of cognitive profiles is limited. The distribution of index scores deviated significantly from the normal distribution in the ASD group, which partly reflects the heterogeneous nature of ASD. The underlying causes of the formation of the observed distribution patterns should be further investigated in future studies.

## Abbreviation:

ASD: Autism Spectrum Disorder
ADHD: Attention Deficit/Hyperactivity Disorder
WAIS: Wechsler Adult Intelligence Scale
DSM: Diagnostic and Statistical Manual of Mental Disorders
PDD-NOS: Pervasive Developmental Disorder-Not Otherwise Specified
VCI: Verbal Comprehension Index
WMI: Working Memory Index
POI: Perceptual Organization Index
PSI: Processing Speed Index
FSIQ: Full Scale Intelligence Quotient
AUC: Area under Curve
ROC: Receiver Operator Characteristics
BIC: Bayesian Information Criteria
GMM: Gaussian Mixture Modelling
CI: Confidence Interval
